# DNA Methylation on N6-Adenine Regulates the Hyphal Development during Dimorphism in the Early-Diverging Fungus *Mucor lusitanicus*

**DOI:** 10.3390/jof7090738

**Published:** 2021-09-08

**Authors:** Macario Osorio-Concepción, Carlos Lax, Eusebio Navarro, Francisco E. Nicolás, Victoriano Garre

**Affiliations:** Departamento de Genética y Microbiología, Facultad de Biología, Universidad de Murcia, 30100 Murcia, Spain; macario.osorio@um.es (M.O.-C.); carlos.lax@um.es (C.L.); sebi@um.es (E.N.)

**Keywords:** mucormycosis, AlkB, demethylase, virulence, dimorphism, protein kinase A, hyphae development, *Mucor*

## Abstract

The epigenetic modifications control the pathogenicity of human pathogenic fungi, which have been poorly studied in Mucorales, causative agents of mucormycosis. This order belongs to a group referred to as early-diverging fungi that are characterized by high levels of N6-methyldeoxy adenine (6mA) in their genome with dense 6mA clusters associated with actively expressed genes. AlkB enzymes can act as demethylases of 6mA in DNA, with the most remarkable eukaryotic examples being mammalian ALKBH1 and *Caenorhabditis elegans* NMAD-1. The *Mucor lusitanicus* (formerly *M. circinelloides* f. *lusitanicus*) genome contains one gene, *dmt1*, and two genes, *dmt2* and *dmt3*, encoding proteins similar to *C. elegans* NMAD-1 and ALKBH1, respectively. The function of these three genes was analyzed by the generation of single and double deletion mutants for each gene. Multiple processes were studied in the mutants, but defects were only found in single and double deletion mutants for *dmt1*. In contrast to the wild-type strain, *dmt1* mutants showed an increase in 6mA levels during the dimorphic transition, suggesting that 6mA is associated with dimorphism in *M. lusitanicus*. Furthermore, the spores of *dmt1* mutants challenged with macrophages underwent a reduction in polar growth, suggesting that 6mA also has a role during the spore–macrophage interaction that could be important in the infection process.

## 1. Introduction

Mucormycosis is an invasive and emerging infection produced by opportunistic fungi of the order Mucorales [1,2]. The infection is characterized by blood vessel invasion and thrombosis formation, concluding in tissue necrosis, facilitating fungal spreading [3]. In addition to patients with pathologies, such as diabetes and an impaired immune system, healthy individuals are also the most at risk for fungal infection [4]. Mucormycosis has been increasingly described in patients COVID-19 associated with uncontrolled diabetes and steroid treatment [5,6]. This concerning scenario caused by the high mortality rates of mucormycosis (up to 90%) has provoked an urgent need to study this lethal infection [7]. Therefore, identifying essential components to the development of mucoralean fungi could contribute to the design of new specific and effective antifungal therapies. In study of the mucormycosis-causing fungi, *M. lusitanicus* (formerly *M. circinelloides* f. *lusitanicus*) [8] has become an excellent genetic model to elucidate the molecular mechanisms of the infection process in the host. The growing development of new molecular and genetic strategies to manipulate the *M. lusitanicus* genome has enabled the discovery of diverse virulence processes important for establishing infection, such as iron assimilation, dimorphism, and the RNA interference mechanism (RNAi) [9,10]. The dimorphism of some Mucorales species consists of the ability to change the growth form between yeast and hyphae, a process regulated by environmental conditions [10,11]. In aerobic conditions, *M. lusitanicus* exhibits filamentous growth, while in an anaerobic environment, it grows exclusively as yeast [12]. This morphological change of Mucorales represents a crucial mechanism for its survival in the host. Mucorales spores can remain latent through the inhibition of phagosome maturation and represent the main form of dissemination, while hyphal growth constitutes the main form of invasion for the establishment of infection in the host. The dimorphic transition of Mucorales gives it the ability to evade and overcome the host defense system to cause mucormycosis [10,13].

Recent studies revealed a connection between the calcineurin pathway, dimorphism, and the virulence of *M. lusitanicus*. Calcineurin is a heterodimeric Ca^2+^/calmodulin-dependent phosphatase constituted by a regulatory (CnbR) and a catalytic subunit (CnaA), regulating the activity of transcription factors through dephosphorylation [12]. The CnbR and CnaA deletion from Calcineurin considerably affect the dimorphic transition [14,15]. Strains blocked in filamentous growth show high virulence, while strains that exclusively grow as yeast are less virulent [14,15]. Other proteins, such as cAMP-dependent protein kinase A (PKA), also mediate the dimorphism and virulence of *M. lusitanicus* [16].

However, the pathogenicity mechanisms used by Mucorales to invade the host and develop the infection have not been completely characterized. In this context, understanding how epigenetic modifications in nucleic acids and proteins influence essential functions in diverse biological processes could be powerful knowledge to obtain effective treatments. For instance, in the human pathogen *Candida albicans*, epigenetic modifications control diverse virulence factors, including antifungal resistance, host biofilm formation, and switch from yeast to hyphae [17,18,19]. DNA methylation is one of these covalent modifications and can be incorporated in DNA as a result of lesions induced by alkylating agents or catalyzed by specific methylases proteins. This epigenetic mark is associated with diverse cell processes in prokaryotes and eukaryotes [20,21]. C5-methylcytosine (5mC) is the most frequent form of DNA methylation in eukaryotes organisms, which involves epigenetic memory maintenance, the regulation of gene expression, transposon inactivation, and genomic imprinting [21,22]. Another form of DNA methylation is N6-methyldeoxyadenine (6mA), initially found in prokaryotic organisms, which has been recently identified in several eukaryotes as being involved in processes such as the regulation of gene expression and transposons, tumorigenesis, stem cell differentiation, and mismatch repair [23,24]. High levels of 6mA have been detected in early diverging fungi, including Mucorales, suggesting that it plays an important role in the biology of these fungi [25]. Genome analysis of several fungi species revealed early fungi contain up to 2.8% of methylated adenines, contrary to the tested higher fungi and other eukaryotes containing 6mA between 0.048 and 0.21%, respectively [25]. The analysis also revealed that 6mA occurs in the ApT dinucleotide of both DNA strands. Most of the 6mA marks are concentrated in methylated adenine clusters (MACs), which are localized around the promoter regions of transcriptionally active genes, indicating that they could be relevant for gene expression regulation [25]. In eukaryotes with elevated 6mA levels in DNA such as *Chlamydomonas reinhardtii* and the ciliates *Oxytrichia trifallax* and *Tetrahymena thermophila*, 6mA distribution also prevails in the ApT sites close to the transcription start sites of active genes [26,27,28]. The recent discovery of proteins responsible for removing methyl groups of 6mA indicates that DNA adenine methylation is a dynamic modification [29]. The demethylation of DNA adenine is accomplished by the activity of 2-oxoglutarate/Fe (II)-dependent dioxygenases enzymes (AlkB), which are conserved from prokaryotes to eukaryotes [30,31]. Bacterial genomes contain between one and three AlkB enzymes important in restoring DNA lesions, such as N1-Methyladenine (1mA), N3-methylcytosine (3mC), and N3-methylthymine (3mT), induced by alkylating agents [32,33,34]. Notably, a variety of species of eukaryotes, including mammals, yeasts, and plants, contain bacterial AlkB homologs (ALKBHs), with the conserved AlkB domain indicating potential functions in various cellular processes [35,36,37]. Humans and mice contain nine ALKBHs, designated as AlKBH1-8 and FTO (fat mass and obesity-associated), localized in different cell compartments [35,38,39]. Like bacterial AlkB enzymes, each member of the ALKBH family preferentially recognizes and demethylates a type of methylation from DNA or RNA [35]. In mammals, AlKBH proteins appear to be associated with genomic stability maintenance, methylated nucleotide repair, and the inhibition of tumor development [40,41]. In recent years, the biological function of DNA and RNA demethylation mediated by ALKBH1 has been extensively studied [42]. It has been found that human ALKBH1 (hALKBH1) shows preference towards the substrates 5-methylcytosine (5mC), 3-methylcytidine (3mC), 6mA, 1-methyladenosine (1mA), suggesting a hALKBH1 role in gene regulation, transduction, and mitochondrial activity [43]. The hALKBH1 overexpression and the consequent decrease in 6mA levels promote the proliferation, migration, and invasion of cancer cells, indicating 6mA functions in the tumorigenesis inhibition [44,45]. In mice, the ALKBH1 absence causes alterations in development and neuronal differentiation [46,47]. In *C. elegans,* from five ALKB proteins found, only one of them shows demethylase activity to 6mA from DNA. The deletion of N6-methyl adenine demethylase 1 (*nmad*-1) results in sterile mutants and presents a high accumulation of 6mA [23]. Recent studies revealed that NMAD-1 also controls DNA replication and repair in the meiosis phase in this organism [48]. On the other hand, unlike *E. coli* ALKB, the two AlkB homologs found in the *Schizosaccharomyces pombe* genome, Ofd2 and Abh1, do not have activity-repairing DNA lesions [49].

In the present study, we investigated the biological role of three AlkB proteins of *M. lusitanicus* with similarity to well-known DNA 6mA demethylases in the physiology of the fungus. They were named Dmt1, Dmt2, and Dmt3. Dmt2 and Dmt3 are closely related to human and mouse ALKBH1, while Dmt1 presents high similarity to N6-methyl adenine *demethylase* 1 (NMAD-1) of *C. elegans*. The deletion of these genes revealed that only *dmt1* is important for the appropriate dimorphic shift from yeast to mycelium and polar growth during the spore–macrophage interaction in *M. lusitanicus*. In addition, 6mA levels during the dimorphic transition were altered in *dmt1* deletion mutants, suggesting that 6mA directly or indirectly regulates dimorphism in this fungus.

## 2. Materials and Methods

### 2.1. Fungal Strains and Culture Conditions

*Mucor lusitanicus* CBS277.49 [50] and the derived strain MU636 [51], a leucine auxotroph, were used as wild-type strains throughout this research. Strain MU402 [52], a uracil and leucine auxotroph, was used as a recipient strain during genetic transformation to generate the *dmt* mutants.

All strains generated in this work are listed in Table S1. *M. lusitanicus* was grown in yeast extract peptone glucose (YPG; 3 g/L yeast extract, 10 g/L peptone, 20 g/L glucose, 15 g/L agar) medium agar plates or liquid, pH 4.5, to evaluate the sporulation, radial growth, and yeast to hyphae transition. The experiments to examine the effect of sodium dodecyl sulfate (SDS), DNA-damaging agents, ultraviolet light, and prooxidants in *M. lusitanicus* were performed on Yeast Nitrogen Base (YNB; 1.5 g/L ammonium sulfate, 1.5 g/L glutamic acid, 0.5 g/L yeast nitrogen base without amino acids and ammonium sulfate, 10 g/L glucose, 15 g/L agar) medium, pH 3, supplemented with 1 mL of thiamine (1 mg/mL) and niacin (1 mg/mL). Culture media were supplemented with uridine (200 mg/L) or leucine (20 mg/L) when necessary for auxotrophy complementation.

### 2.2. Disruption of dmt Genes and Generation of Complemented Strains

The generation of protoplasts and the genetic transformation by electroporation were performed according to the previously described protocol [53].

For the single deletion of *M. lusitanicus dmt* genes, we constructed recombinant fragments by overlapping PCR. Constructs containing a 2 kb sequence of the *pyrG* selectable marker were flanked by 1 kb sequence up- and downstream of each *dmt* gene. Up- and downstream fragments of each *dmt* gene and *pyrG* gene were PCR amplified and subjected to overlap PCR using specific primers (Table S2) to obtain the deletion fragment. These constructs were used to genetically transform strain MU402 (*pyrG−*, *leuA−*) by electroporation to delete the target locus by homologous recombination [53]. Homokaryotic transformants (MU1316, MU1317, MU1318, MU1319, MU1320, and MU1321) were selected after several vegetative cycles on minimal medium with casamino acids (MMC), pH 3.2, supplemented with niacin (1 mg/mL) and thiamine (1 mg/mL) [54]. Gene deletion and homokaryosis were confirmed by Southern blot hybridization using specific probes.

To generate double-knockout mutants, deletion fragments were generated as detailed previously. The *leuA* gene (3.2 kb in length) was surrounded by 1 kb up- and downstream sequences of the *dmt2* and *dmt3* genes that allowed the gene replacement by double homologous recombination. These constructs were used for the genetic transformation of the recipient strain MU1317 for the generation of *dmt1*Δ/*dmt2*Δ mutant (MU1325 and MU1326) and *dmt1*Δ/*dmt3*Δ mutant (MU1322 and MU1324). The construct employed for *dmt3* deletion was also used for the transformation of mutant MU1318 to obtain the *dmt2*Δ/*dmt3*Δ double mutant (MU1328 and MU1330). Up- and downstream regions of *dmt* and *leuA* genes that constituted each of the constructs for gene deletion were amplified with specific primers (Table S2). Transformants were selected after several cycles of vegetative growth on YNB medium, pH 3.2, supplemented with niacin (1 mg/mL) and thiamine (1 mg/mL) [55].

To complement the MU1317 by reintroducing the *dmt1* wild-type allele, a construct was designed as follows: the *dmt1* open reading frame (ORF) including 1 kb 5′- and 3’- flanking regions were amplified from genomic DNA of *M. lusitanicus* CBS277.49 employing specific primers bearing *Xho*I and *Sac*II restriction sites (Table S2). The *dmt1* fragment was cloned in the corresponding sites into the pMAT1476 plasmid [56], which contains the *leuA* selectable marker flanked by 5´- and 3´- ends of the *carRP* gene. The pMAT2250 plasmid constructed was digested with the *Sac*I and *Pvu*I restriction enzymes to release the whole construct that included the *leuA* cassette, *dmt1* allele, and *carRP* flanking sequences. The pMAT1476 plasmid was also digested with the *Sma*I enzyme to release the cassette only containing the *leuA* gene surrounded by *carRP* flanking sequences. The constructs were used to transform the MU1317 protoplast for its integration at the *carRP* locus. The transformants with correct integration developed colonies with white patches because of the deletion of the *carRP* gene. After several vegetative cycles on YNB medium [55], pH 3.2, supplemented with niacin (1 mg/mL) and thiamine (1 mg/mL), completely white colonies were obtained (MU1331 and MU1334), as a result of the homokaryosis confirmed by PCR (Table S1).

### 2.3. Southern Blot

For Southern blot hybridization, specific probes amplified from gDNA that discriminate between the wild-type and mutant alleles were obtained by PCR amplification using the following specific primers: Udmt1F/Ddmt1-pyrGR, Udmt2F/Udmt2-pyrGR, and Udmt3F/Ddmt3-pyrGR for the genes *dmt1*, *dmt2,* and *dmt3*, respectively (Table S2). DNA probes were labeled using α-^32^P dCTP employing Read-To-Go Labeling Beads (GE Healthcare Life Science, Chicago, IL, USA). A total of 1 µg of gDNA digested with restriction enzymes appropriated and separated by electrophoresis was transferred to an Amersham Hybond XL membrane (GE Healthcare Life Sciences) for the hybridization.

### 2.4. Experiments of Yeast to Hyphae Transition

For the induction of the yeast to hyphae transition, 1 × 10^6^/mL fresh spores of different strains of *M. lusitanicus* were inoculated in 50 mL conical tubes filled with liquid YPG media pH 4.5 freshly autoclaved and sealed with parafilm. The cultures were incubated at 26 °C without shaking for 24 h to yeast induction. A total of 15 mL of the previous culture was poured into a 50 mL flask and placed in a shaker at 250 rpm and 26 °C for 2.5 h for hyphal induction. The yeast and hyphae formed were observed by optical microscopy and photographed at 20 or 40× magnification. The percentage of cells with hyphae formation was calculated from 200 total cells. The polarity index of 50 yeasts was measured with hyphae emergency using ImageJ software from micrographs taken 2.5 h after hyphal induction. The polarity index corresponded to the ratio between cell length and width. The statistical significance of each experiment was calculated using the Tukey test ANOVA (*p* < 0.001) and the unpaired *t*-test (*p* < 0.05).

### 2.5. In Vitro Host-Pathogen Interaction Assays

To evaluate the survival of the *dmt* mutants in vitro host–pathogen interactions, fungal spores were co-inoculated with mouse macrophages J774A.1 in a ratio of 1.5 spores per macrophage following the previously described protocol [57] in Leibovitz L-15 Medium at 37 °C (Biowest, Minneapolis, MN, USA) containing 10% fetal bovine serum (FBS) and 1% penicillin/streptomycin (Gibco). After 30 min, the cells were washed three times with 1× phosphate-buffered saline (PBS) to remove non-phagocytosed spores and placed at 37 °C for 5.5 h. At that time, micrographs of spore germination into macrophages were used to determine the polarity index from 50 spores, as previously described [57]. To analyze spore viability after phagocytosis, the same macrophage cultures were treated with 0.1% NP-40 cell lysis buffer to release the phagocytosed spores. A total of 10 µL of each sample was seeded on MMC agar plates, pH 3.2, and incubated at 26 °C for 48 h. The growth and development of healthy colonies was examined by visual inspection. Spores without macrophages cultured in L-15 medium under the same conditions were employed as a control.

### 2.6. RNA Extraction and cDNA Synthesis

The RNA extraction was carried out from samples collected several times (0 min, 30 min, 1 h, and 2 h) during the induction of yeast to hyphae transition by the TRIzol method according to the supplier recommendation (Invitrogen, Waltham, MS, USA). RNA concentration and quality were determined using Qubit fluorimeter (Invitrogen) and agarose gel electrophoresis, considering the 28S/18S rRNA ratio, respectively. A total of 1 µg of total RNA was treated with TURBO DNase (Thermo Fisher, Waltham, MS, USA) and was used for cDNA synthesis, employing the iScript cDNA Synthesis Kit (Bio-Rad, Hercules, CA, USA) following the supplier recommendations. The cDNA synthesized was used as templated for real-time PCR assays.

### 2.7. RT-qPCR Analysis

The transcriptional profile of the *pkaR4* gene was analyzed by qPCR at different times of induction of yeast to hyphae transition (0 min, 30 min, 1 h, and 2 h) with specific primers (Table S2). The gene amplification was carried out in triplicate using a reaction mixture containing 1× Power Sybr Green Master Mix 2× (Applied biosystem, Waltham, MS, USA), 150 nM of *pkaR4*-specific primers, and 100 ng of cDNA templated. The real-time PCR was carried out using the QuantStudio^TM^ 5 real-time PCR system (Applied biosystem) according to the established experiment template in the equipment. The melting curve and non-template controls were also measured to discern non-specific amplifications. The relative expression of *pkaR4* was normalized with the amplification levels of elongation factor 1 alpha gene (*ef-1*) [58] and calculated using the 2^−ΔΔ^*^CT^* method [59].

### 2.8. Analysis of 6mA in DNA by HPLC-MS

Genomic DNA extracted from yeast and mycelia samples collected before (0 h) and after (2 h) dimorphism induction was digested to single nucleosides following previously established procedures [26,60]. A total of 2 μg of gDNA diluted to a total volume of 26 μL with nuclease-free water was heated at 100 °C for 3 min and chilled on ice for 2 min. Treated DNA samples were digested with 1.5 U of DNAse I overnight at 42 °C (ThermoFisher) and 0.001 U of Phosphodiesterase I from *Crotalus adamanteus* venom (Merck, Darmstadt, Germany) to 37 °C for 2 h in 100 mM NH_4_HCO_3_ buffer, pH 7.8. Finally, digested gDNA was treated with 1 U of alkaline phosphatase at 37 °C for 2 h and diluted two-fold with nuclease-free water. Single nucleotides were analyzed using an HPLC-MS system consisting of an Agilent 1290 Infinity II Series HPLC (Agilent Technologies, Santa Clara, CA, USA) equipped with an Automated Multisampler module and a High-Speed BinaryPump, and connected to an Agilent 6550 Q-TOF Mass Spectrometer (Agilent Technologies, Santa Clara, CA, USA) using an Agilent Jet Stream Dual electrospray (AJS-Dual ESI) in the positive mode. Experimental parameters for HPLC and Q-TOF were set in MassHunter Workstation Data Acquisition software (Agilent Technologies, Rev. B.08.00). The nucleosides were quantified using the nucleoside to base ion mass transition of 252.1091 > 136.0638 m/z for dA (C_10_H_13_N_5_O_3_) and 266.1248 > 150.0812 m/z for 6mA (C_11_H_15_N_5_O_3_). The 6mA/dA ratio was calculated based on the concentration of each nucleoside.

### 2.9. Sensitivity Tests

Different concentrations of fresh spore of analyzed strains (10^4^, 10^3^, 10^2,^ and 10 spores) were spotted on YNB agar plates, pH 3, amended with 0.005% sodium dodecyl sulfate (SDS), 0.2% ethyl methane sulphonate (EMS), and 2 mg/mL hydroxyurea (HU).

For hydrogen peroxide (H_2_O_2_) and UV-associated assays, 200 spores were spread on YNB plates, pH 3, supplemented with 5 mM H_2_O_2_, or exposed to 10 mJ/cm^2^ UV (254 nm), respectively. All the plates were incubated at 26 °C by 48 h to evaluate the *M*. *lusitanicus* ability to develop colonies in all conditions tested and estimate the survival percentage from total cells inoculated.

## 3. Results

### 3.1. Genes of M. lusitanicus Encoding Putative 6mA Demethylases

We searched homologs of eukaryotic enzymes with demonstrated ability to remove methyl groups in the *M. lusitanicus* genome (CBS277.49 v2.0; https://mycocosm.jgi.doe.gov/Mucci2/Mucci2.home.html (accessed on 8 Octerber 2018)). A blast tool using *C. elegans* NMAD-1 [23] and mouse ALKBH1 [43] identified one (ID 85076, scaffold 06:396232-397096) and two similar putative proteins in the fungus, respectively (ID 106998, scaffold 02:2304316-2305722; and ID 115786, scaffold 11:718495-719875). The deduced amino acid sequence of the three identified proteins contained the conserved domain 2-oxoglutarate and Fe (II)-dependent dioxygenase (2OG-FeII_Oxy_2), similar to the domain identified in known AlkB enzymes. The presence of this domain and their similarity with characterized 6mA demethylases suggested that they could be involved in removing methyl groups in 6mA [31,61], regulating cellular processes of the fungus in this way. The genes encoding these hypothetical proteins were named *dmt1* (ID 85076)*, dmt2* (ID 106998)*,* and *dmt3* (ID 115786).

### 3.2. Generation of Single and Double dmt Knockout Mutants in M. lusitanicus

To analyze the function of the three *dmt* genes in *M. lusitanicus*, single and double mutants of each *demethylase* were generated by double homologous recombination. For single-deletion mutants, disrupting fragments containing *pyrG* selectable marker were used for genetic transformation and the replacement of each *dmt* gene in the strain MU402 (*pyrG*−, *leuA*−) (Figure S1). After several cycles of vegetative growth on selective media to isolate homokaryons, two independent transformants of each knockout experiment were selected and analyzed by Southern blot hybridization. All transformants selected were homokaryons for the mutant allele because they only showed the hybridization fragment corresponding to the mutant allele (Figure S1). These single knockout mutants were named as follows: MU1316 and MU1317 had a deletion of the *dmt1* gene, MU1318 and MU1319 had a deletion of the *dmt2* gene, and MU1320 and MU1321 had a deletion of the *dmt3 gene.*

Additionally, double knockout mutants in the *dmt* genes were generated from single deletion mutants (*pyrG*+, *leuA−*) by using recombinant fragments containing the selection marker *leuA*, which complement their leucine auxotrophy (Figure S2). Strain MU1317 (*dmt1*Δ*::pyrG*+, *leuA−*) was used as the recipient for the replacement of *dmt2* and *dmt3*. Two independent homokaryotic transformants were selected that carried the deletion of *dmt1* and *dmt2* (MU1325 and MU1326) (Figure S2A) and another two with a deletion of *dmt1* and *dmt3* (MU1322 and MU1324) (Figure S2B). To generate the *dmt2*Δ/*dmt3*Δ knockout mutant, strain MU1319 (*dmt2*Δ*::pyrG*+, *leuA−*) was used for genetic transformation with recombinant fragment targeting the *dmt3* gene (Figure S2C). Two independent homokaryons (MU1328 and MU1330) were selected. The correctness of the deletions and homokaryosis were confirmed by PCR (Figure S2A–C). Both independent single and double deletion mutants were used to analyze the biological role of *M. lusitanicus dmt* genes in different conditions. However, in most experiments, just one member of each mutant pair was analyzed when no differences with the wild-type strains were found.

### 3.3. The dmt1 Is Involved in the Hyphae Formation during the Yeast to Mycelium Transition

To investigate whether any of the three *dmt* genes play a role in the biology of *M. lusitanicus*, single and double deletion mutants were characterized phenotypically under different growth conditions. Single deletion mutants MU1317 (*dmt1*Δ), MU1318 (*dmt2*Δ), and MU1320 (*dmt3*Δ) showed colonial morphology, mycelial growth, and spore production similar to the wild-type strain MU636 (Figures S3A and S4A,B). Double deletion mutants MU1324 (*dmt1*Δ/*dmt3*Δ) and MU1326 (*dmt1*Δ/*dmt2*Δ) also exhibited parameters similar to the wild-type strain CBS277.49, whereas and MU1330 (*dmt2*Δ/*dmt3*Δ) showed a slightly different colony morphology and 50% reduction in spore production, but wild-type mycelial growth (Figures S3B and S4C,D). Bacterial and mammalian AlkB can restore DNA lesions induced by alkylating agents, which are generated by double-strand breaks and the replication inhibition of DNA [35,62]. Thus, strains were exposed to ethyl methane sulphonate (EMS) and hydroxyurea (HU), which cause double-strand breaks and the replication inhibition of DNA, respectively. *dmt* knockout strains showed no changes in the sensitivity to EMS (0.2%) or HU (2 mg/mL) compared to the wild type strain (Figure S5A,B). Similar behavior was observed in strains exposed to ultraviolet light (UV), while higher doses inhibited the growth of wild-type and mutant strains (Figure S6B). These data demonstrate that none of the three genes by themselves are essential in DNA repair.

The presence of sodium dodecyl sulfate (SDS) in the culture medium to evaluate the integrity of the cell wall affected the growth of both single (Figure S6A) and double mutants, similar to the wild type strain, which indicates that the integrity of the cell wall is not compromised with the deletion of any of the three *dmt* genes. Later, the ability of *M. lusitanicus* to respond to stress conditions generated by hydrogen peroxide (H_2_O_2_) was tested; both the single (Figure S6C) and double knockout mutants presented a response equivalent to the wild-type strain to 5 mM H_2_O_2_. Taken together, these data may suggest that the three *dmt* genes evaluated are not directly associated with the mechanism regulating the mycelial growth, DNA repair, cell wall integrity, and response to oxidative stress. Alternatively, redundancy among the three *dmt* genes could explain the wild-type phenotype of single and double deletion mutants. In fact, redundancy may explain why the deletion of both *dmt2* and *dmt3* provided a reduction in spore production, whereas single knockout mutants showed the wild-type phenotype (Figure S4D).

**Figure 1:**
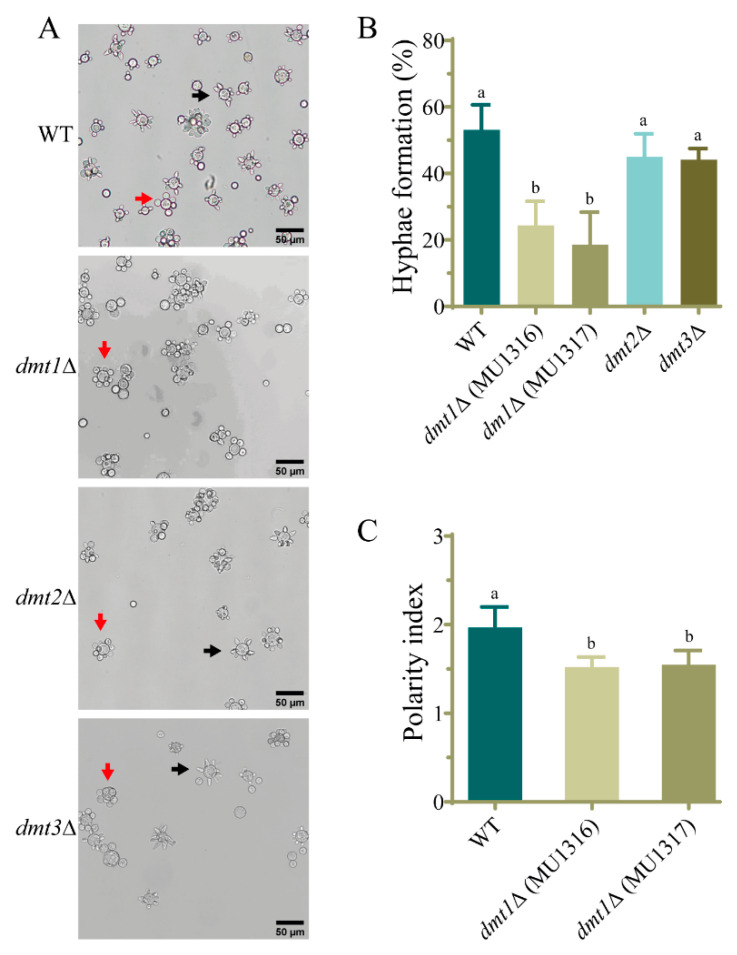
The mutants in the *dmt1* gene of *M. lusitanicus* are affected in the hyphae emergence during yeast to mycelium transition. (**A**) Cells of wild-type strain MU636 (WT), *dmt1*Δ (MU1316 and MU1317), *dmt2*Δ (MU1318), and *dmt3*Δ (MU1320) 2.5 h after induction of hyphal development by transferring to aerobic conditions. The arrows indicate the multi-budded yeasts (red) and cells with hyphae (black). (**B**) The data represent the percentage of hyphae formation 2.5 h after transition induction calculated from 200 total cells. (**C**) Polarity index quantified from 50 yeasts with hyphae emergency after 2.5 h induction. The charts display means ± SD. Different letters above the bars indicate statistically significant differences, while the identical letters denote no significant differences calculated using one-way ANOVA (*p* < 0.001, Tukey test). Three independent experiments were conducted for each strain.

**Figure 2:**
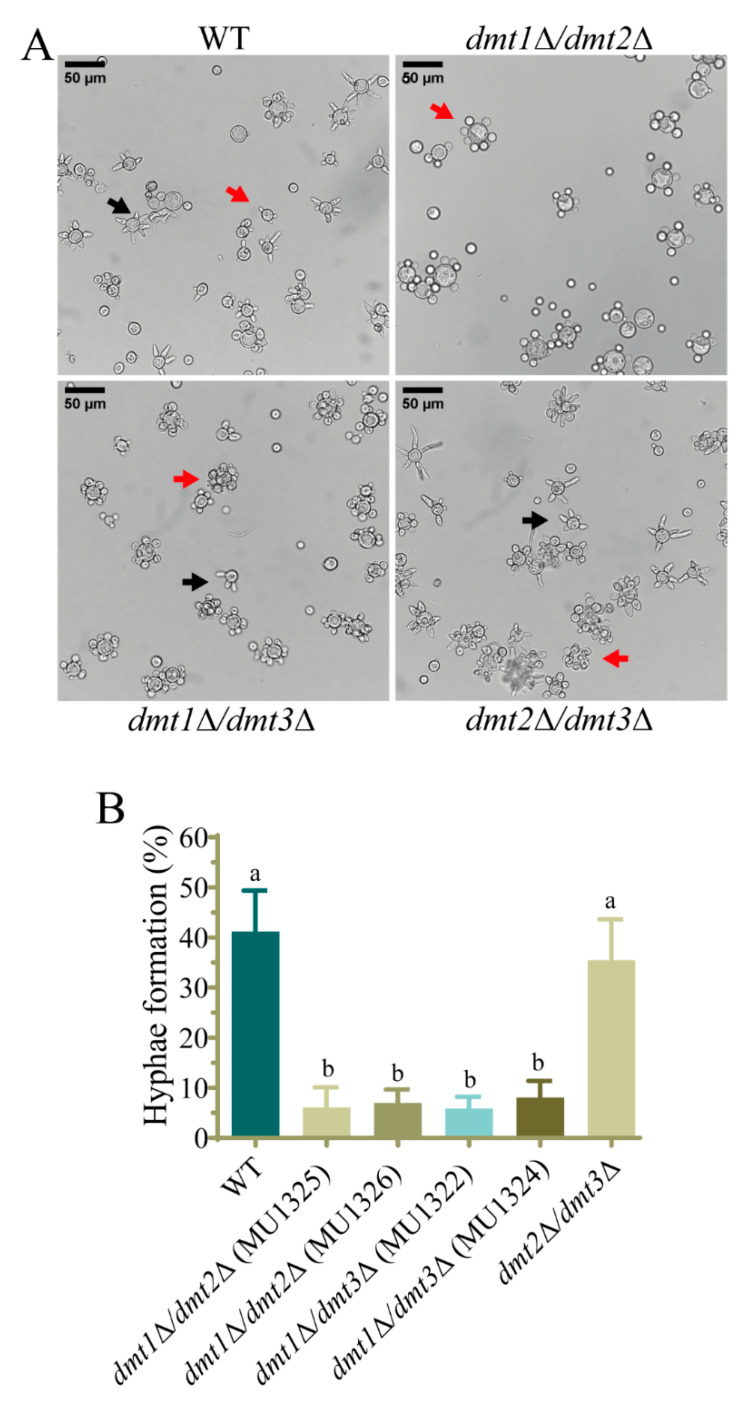
The double mutants having *dmt1* deletion were affected in the hyphae emergence during yeast to mycelium transition. (**A**) Cells of wild-type strain CBS277.49 (WT), *dmt1*Δ/*dmt2*Δ (MU1325 and MU1326), *dmt1*Δ/*dmt3*Δ (MU1322 and MU1324), and *dmt2*Δ/*dmt3*Δ (MU1330) 2.5 h of growth after induction of hyphae formation by transferring to aerobic conditions. The arrows indicate the multi-budded yeasts (red) and cells with hyphae (black). (**B**) Percentage of yeasts with developed hyphae in wild type strain (CBS277.49) and the indicated double knockout mutants calculated from 200 total cells. The charts display means ± SD. Different letters above the bars indicate statistically significant differences, while the identical letters denote no statistical differences calculated using one-way ANOVA (*p* < 0.001, Tukey test). Three independent experiments were conducted for each strain.

*M. lusitanicus* has the ability to grow as yeast or hyphal depending on the environmental conditions, and this is closely related to its virulence [14]. Thus, the yeast to hyphae transition was tested in the single and double *dmt* mutants. For this, the spores from *dmt* mutants were inoculated in YPG medium in anaerobic conditions to induce yeast growth, and after 24 h they were aerated to stimulate the yeast to hyphae transition. The formation of hyphae was analyzed at different times (2.5, 3.5, and 5 h) after aeration. Similarly to the wild-type strain, deletion mutants in the *dmt* genes showed markedly multi-budded yeasts in anaerobic conditions, but when the transition from yeast to hyphae was induced, the two independent *dmt1* mutants, MU1316 and MU1317, showed a smaller number of yeast cells producing hyphae than the wild-type strain at 2.5 h (Figure 1A). At longer times in the presence of air, no differences were observed, indicating that *dmt1* deletion provokes a delay in the yeast to hyphae transition (Figure 1A,B). This phenotype was confirmed in the double mutants MU1326 (*dmt1*Δ/*dmt2*Δ) and MU1324 (*dmt1*Δ/*dmt3*Δ) harboring a deletion of *dmt1*, but not in double deletion mutants for *dmt2* and *dmt3* (MU1330) (Figure 2A,B). We determined the polarity index, the ratio between cell length and width, in yeast with hyphae emergency to quantify polar growth and validate the delay in the yeast-to-mycelium transition. Interestingly, strains lacking *dmt1* presented an impaired polar growth than that of the wild-type strain at 2.5 h after aeration (Figure 1C), suggesting that *dmt1* is important for the hyphal development of *M. lusitanicus* during the morphologic shift from yeast to hyphae.

**Figure 3:**
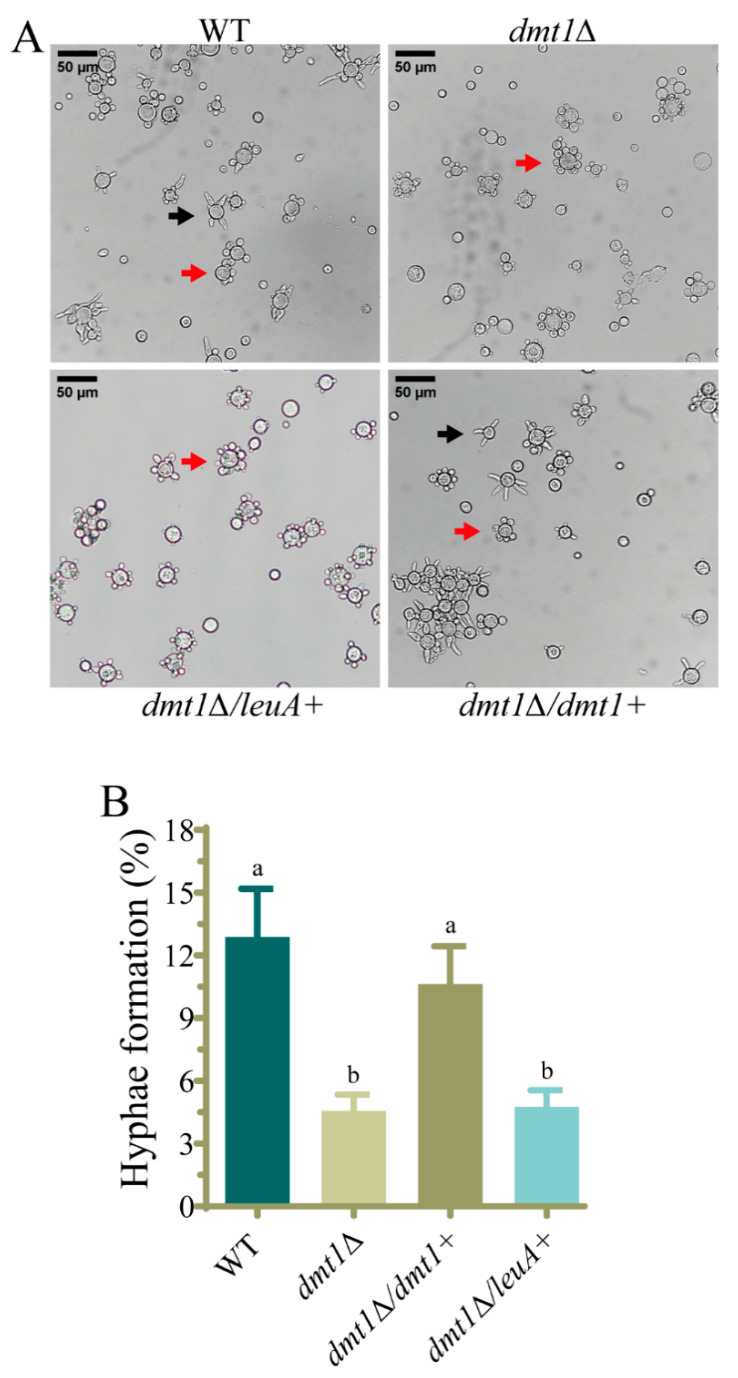
Complementation of *dmt1* mutant with the wild-type allele of *dmt1* gene recovered the ability to develop hyphae. (**A**) Cells of the wild-type strain MU636 (WT), *dmt1*Δ (MU1317), *dmt1*Δ complemented with *dmt1* wild-type allele (MU1331), and *dmt1*Δ complemented with empty cassette (MU1334) 2.5 h after induction of mycelial growth by transferring to aerobic conditions. The arrows indicate the multipolar budding yeasts (red) and cells with germinated hyphae (black). (**B**) Percentage of yeasts with developed hyphae in wild type (WT) strain and the indicated strains calculated from 200 total cells. The charts display means ± SD. Different letters indicate statistically significant differences, while the identical letters denote no significant differences, calculated using one-way ANOVA (*p* < 0.001, Tukey test). Three independent experiments were conducted for each strain.

To test this hypothesis, the strain MU1317 (*dmt1Δ::pyrG+*, *leuA−*) was complemented with the wild-type *dmt1* gene. A construct containing the selective marker *leuA* and wild-type *dmt1* gene flanked by the 5′and 3′ ends of the *carRP* gene was used for the genetic transformation of MU1317 targeting the *carRP* gene, which codes for an enzyme required for carotenoid biosynthesis [63] (Figure S2D). As a control, a similar construct without the *dmt1* gene was also used to transform MU1317 (Figure S2E). Transformants with albino patches, due to the disruption of the *carRP* gene, were grown on a selective medium to obtain homokaryotic strains that were analyzed by PCR to confirm the gene replacement (Figure S2D,E). One homokaryotic strain harboring the wild-type *dmt1* allele (MU1331) and one control strain (MU1334) containing the only selective marker *leuA* were selected for further analysis (Figures S2D,E and S3C). The strain harboring the wild-type *dmt1* allele recovered the ability to develop hyphae after transition induction. In contrast, the control strain, MU1334, exhibited a similar phenotype to the *dmt1* mutant (Figure 3A,B), supporting the idea that *dmt1* plays a key role in the dimorphic transition from yeast to mycelium of *M. lusitanicus.*

### 3.4. The dmt1 Is Important for the Appropriated pkaR4 Transcription during the Transition from Yeast to Hyphae

**Figure 4:**
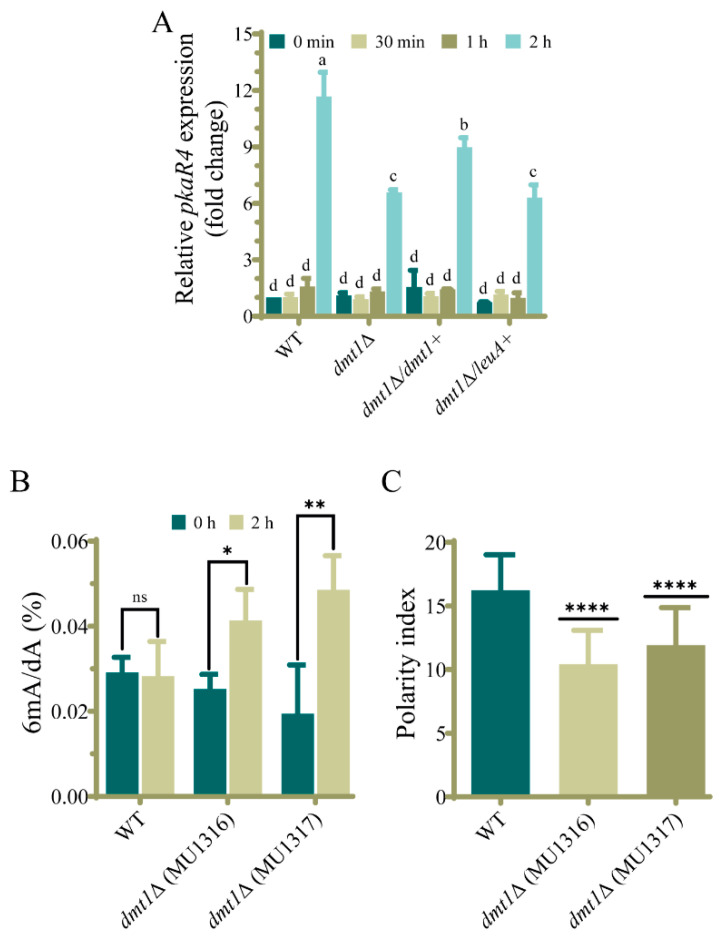
The gene *dmt1* is required for appropriate expression of the *pkaR4* gene during yeast to hyphae transition. (**A**) mRNA levels of *pkaR4* were determined by RT-PCR from total RNA isolated from MU636 (WT), *dmt1*Δ (MU1317), *dmt1*Δ complemented with *dmt1* wild-type allele (MU1331), and *dmt1*Δ complemented with empty cassette (MU1334) at different times (0 min, 30 min, 1 h, and 2 h) after induction of transition from yeast to mycelium. The amplification levels of *ef-1* were used to normalize the relative expression of *pkaR4* using the 2^−ΔΔ^*^CT^* method. (**B**) Levels of 6mA, determined by HPLC-MS analysis, in genomic DNA isolated from yeast cells before (0 h) and 2 h after transferring them to aerobic conditions of the wild-type strain MU636 (WT) and *dmt1* mutants (MU1316 and MU1317). (**C**) Polarity index of the wild-type strain MU636 (WT) and *dmt1* mutants MU1316 and MU1317 measured 5.5 h after phagocytosis by mouse macrophages line J774A.1. The polarity index was calculated from 50 phagocytosed spores. The charts display means ± SD. Different letters above the bars indicate statistical significance, while the identical letters denote no significant differences (*p* < 0.05, Tukey test). Asterisks denote a significant difference calculated using one-way ANOVA (**** *p* < 0.0001) and unpaired *t*-test (* *p* < 0.05, ** *p* < 0.001; ns, not significant).

To further support the function of *dmt1* in dimorphism, we analyzed the mRNA accumulation of *pkaR4*, a gene encoding one regulatory subunit of cAMP-dependent protein kinase A (PKA) required for germ tube emergence in aerobic conditions [16]. To that end, yeast cells of *dmt1*Δ and the complemented strain were transferred from anaerobic to aerobic conditions to induce hyphal growth, and samples were taken at different times (0 min, 30 min, 1 h, and 2 h). In the early times after induction of the dimorphic transition (30 min and 1 h), *pkaR4* transcript levels remained unaltered in comparison to the yeast form (0 min) in all tested strains, but after 2 h, *pkaR4* mRNA levels increased 11.5-fold in the wild-type strain (Figure 4A). Interestingly, at this time, the *pkaR4* transcript levels in the *dmt1*Δ strain were significantly lower (6.5-fold increase) (Figure 4A), suggesting that *dmt1* regulates *pkaR4* expression. This result was further confirmed with the quantification of *pkaR4* expression in the strain that ectopically expressed *dmt1*, because a partial recovery of the *pkaR4* expression (8.3-fold increase) was observed, compared to its control (MU1334), in which only the *leuA* marker displays similar induction to the *dmt1* mutant (Figure 4A). In consequence, these results suggest that *dmt1* controls germ tube emergence in the dimorphic transition of *M. lusitanicus* by regulating the expression of *pkaR4*.

### 3.5. Deletion of dmt1 Alters 6mA Levels during Yeast to Hyphae Transition

The protein encoded by *dmt1* is highly similar to AlkB proteins from eukaryotes, particularly to the *C. elegans* NMAD-1, which demethylates 6mA in DNA. Therefore, we hypothesized that Dmt1 could also remove this epigenetic mark in DNA. Thus, we measured 6mA levels in yeasts and 2 h after the transition to aerobic conditions by HPLC-MS in *dmt1* mutants and the wild-type strain. The genomic 6mA levels were similar in both conditions in the wild-type strain (Figure 4B), indicating that 6mA is maintained during the dimorphic transition. Interestingly, and in contrast to the wild-type strain, both *dmt1* mutants showed an increase in the 6mA levels only after the transition (Figure 4B), suggesting that the *dmt1* gene is required to sustain the 6mA levels during the transition from yeast to hyphae.

### 3.6. dmt1 Regulates Germination in the Interaction of Spores with Macrophages In Vitro

Because the *dmt1* mutants showed a delay in the hyphae formation during yeast to hyphal transition, we hypothesized that this phenomenon could impact the ability of *M. lusitanicus* spores to survive during the macrophage interaction and escape phagocytosis. To support this hypothesis, fresh spores of *dmt1* mutants MU1316 and MU1317 were co-inoculated with mouse macrophages in a cell culture medium. The *dmt1* deletion did not affect the ability of *M. lusitanicus* to survive the cytotoxic environment of the macrophage’s phagocytosis, because spores collected after 5.5 h of interaction developed healthy colonies similar to the wild-type strain. However, the germination capacity inside the phagosome was slightly affected during the co-culture with macrophages, as the polarity index of *dmt1* mutants was lower than that of the wild-type strain (Figure 4C). This delay in germination suggests that *dmt1* plays a role in this process and can be important in the course of the infection.

## 4. Discussion

A high density of 6mA has been associated with active transcription in early-diverging fungi [25], suggesting enzymes that modify its levels in the genome could regulate cell processes by regulating transcription in this group of fungal lineages, which includes Mucorales. In the present study, we analyzed the function in *M. lusitanicus* of three genes, *dmt1*, *dmt2*, and *dmt3*, encoding putative 6mA demethylases because of their similarity with known eukaryotic 6mA demethylases. Gene products of *dmt2* and *dmt3* were closely related to human and mouse ALKBH1, while *dmt1* was similar to *C. elegans* NMAD-1. The NMAD-1 deletion causes a high accumulation of 6mA in *C. elegans*, indicating that this enzyme is the main demethylase of this epigenetic mark [23,64]. A similar event has been reported to mammalian ALKBH1 [43]. The three Dmt protein sequences include an AlkB domain found in ALKB enzymes responsible for removing the methyl group of DNA [23,64], playing important roles in several biological processes in both prokaryotes and eukaryotes [31]. These findings suggested that the products of some *dmt* genes could act in a similar way to NMAD-1 and ALKBH1 and regulate cellular processes in *M. lusitanicus*.

Phenotypes previously related to AlkB proteins such as sporulation, growth, and DNA repair [35,62,65] were unaltered in mutants with a deletion in one or two *dmt* genes, except the double knockout mutant in *dmt2* and *dmt3*, which showed a slight alteration of the colony morphology and a reduction in sporulation. These results could be explained by the fact that these genes are unlinked to the studied processes or their functions overlap, as it seems to occur for *dmt2* and *dmt3* in sporulation. Interestingly, the deletion of *dmt1* either alone or in combination with *dmt2* or *dmt3* leads to a delay in germination tube emission during the dimorphic transition from yeast to mycelium. In contrast, the single deletion of *dmt2* and *dmt3* or the deletion of both genes produced a wild-type phenotype (Figure 1 and Figure 2), suggesting that the defect in dimorphism was exclusively due to the absence of *dmt1*. This hypothesis was further supported by reversing the defect in strains that expressed *dmt1* wild-type allele ectopically (Figure 3). These findings evidence a central role of this *dmt1* in the control of morphogenesis in *M. lusitanicus*, probably through the regulation of the expression of genes involved in the transition from yeast to mycelium. This regulation could be mediated by modifying the 6mA pattern of genes involved in the transition. Therefore, we measured the total 6mA levels in the wild-type strain and *dmt1* mutants during the transition from yeast to mycelium. In contrast to the wild-type strain, which maintained similar 6mA levels during the transition, the *dmt1* mutants showed an increase after mycelial growth was induced (Figure 4B), suggesting that there was an over-methylation in the absence of *dmt1* that could affect the expression of some genes involved in the dimorphism. These results hint at *dmt1* encoding a 6mA demethylase that regulates the expression of some critical genes during the transition from yeast to mycelium by lowering their 6mA levels during the transition.

In recent years, mucormycosis has become one of the main invasive fungal infections due to its rapid growth and its ability to shift morphologically during infection [9]. The substantial contribution of the morphological shift from yeast to hyphae of *M. lusitanicus* to its virulence and pathogenesis [66] makes dimorphism a promising target for the treatment of mucormycosis and the design of new antifungal drugs [14,15]. A few proteins regulating dimorphism have been characterized, with the most remarkable being calcineurin and PKA [14,15,16]. Strains lacking the calcineurin regulatory B subunit (CnbR) remain in the yeast form and show reduced virulence [14,15], while strains lacking the catalytic A subunit (CnaA) remain in the filamentous phase and show high virulence [14]. In addition, this fungus has four genes encoding regulatory subunits of PKA with different roles in physiology [16]. One of these genes, *pkaR4,* is transcribed exclusively during morphogenesis from yeast to mycelium and plays a critical role in mycelial growth as its deletion prevents mycelial growth, whereas overexpression promotes filamentous growth [16]. As previously described, we found low expression levels of *pkaR4* in yeasts and a sharp increase when filamentous growth started (Figure 4A). Although *dmt1* mutants also showed an increase in *pkaR4* transcripts during the transition, the induction was lower than in the wild-type and complemented strains, suggesting that *dmt1* directly or indirectly regulates *pkaR4* expression. The reduced *pkaR4* expression in *dmt1* mutants could be responsible for the delay in germ tube development during the transition from yeast to mycelium.

In addition to the function of *dmt1* in dimorphism, it is also involved in spore germination during interaction with macrophages because spores of the *dmt1* mutants showed a delay in germination, evidenced by a low polarity index (Figure 4C). *M. lusitanicus* strains exhibiting a low polarity index in the interaction with macrophages also present reduced virulence [57]. Accordingly, the delayed polar growth due to the disruption of the *dmt1* gene could affect the virulence of *M. lusitanicus*. The role of epigenetic modifications in the control of cellular processes, including virulence, has not been studied in early-diverging fungi. Consequently, this work represents the first study describing the function of a gene that modifies the DNA. Further analysis to reveal the gene network mediated by *dmt* regulation could be necessary to understand all the implications of *dmt1* in gene expression regulation.

## Data Availability

Not applicable.

## Supplementary Materials

The following are available online at https://www.mdpi.com/article/10.3390/jof7090738/s1, Figure S1: Gene replacement by homologous recombination of the *dmt* genes in *M. lusitanicus*, Figure S2: Generation of *dmt* double mutants and complementation of strain MU1317 (*dmt1*Δ) with *dmt1* wild-type allele, Figure S3: Phenotypic analysis of *dmt* mutants and complemented strains, Figure S4: Single and double deletion mutants in the *dmt* genes are not affected in the growth and sporulation. Figure S5: Phenotypic analysis of single deletion mutants in the *dmt1*, *dmt2,* and *dmt3* genes exposed to alkylating agents and compounds inhibiting DNA synthesis, Figure S6: Survival analysis of single deletion mutants in the *dmt1*, *dmt2,* and *dmt3* genes exposed to stress generating compounds and DNA damaging agents, Table S1: Mutants generated in this study, Table S2: Primers used in this work.
