# Supplementary material for: DNA Methylation on N6-Adenine Regulates the Hyphal Development during Dimorphism in the Early-Diverging Fungus Mucor lusitanicus
Source: J Fungi (Basel). 2021 Sep 8;7(9):738. doi: 10.3390/jof7090738 (PMC8470550; doi:10.3390/jof7090738)
Supplement: Supplementary file 1 [file jof-07-00738-s001.zip › jof-1330263-supplementary/jof-1330263-revised/Supplementary Information_Osorio-Concepcion et al_Figure S1.pdf]

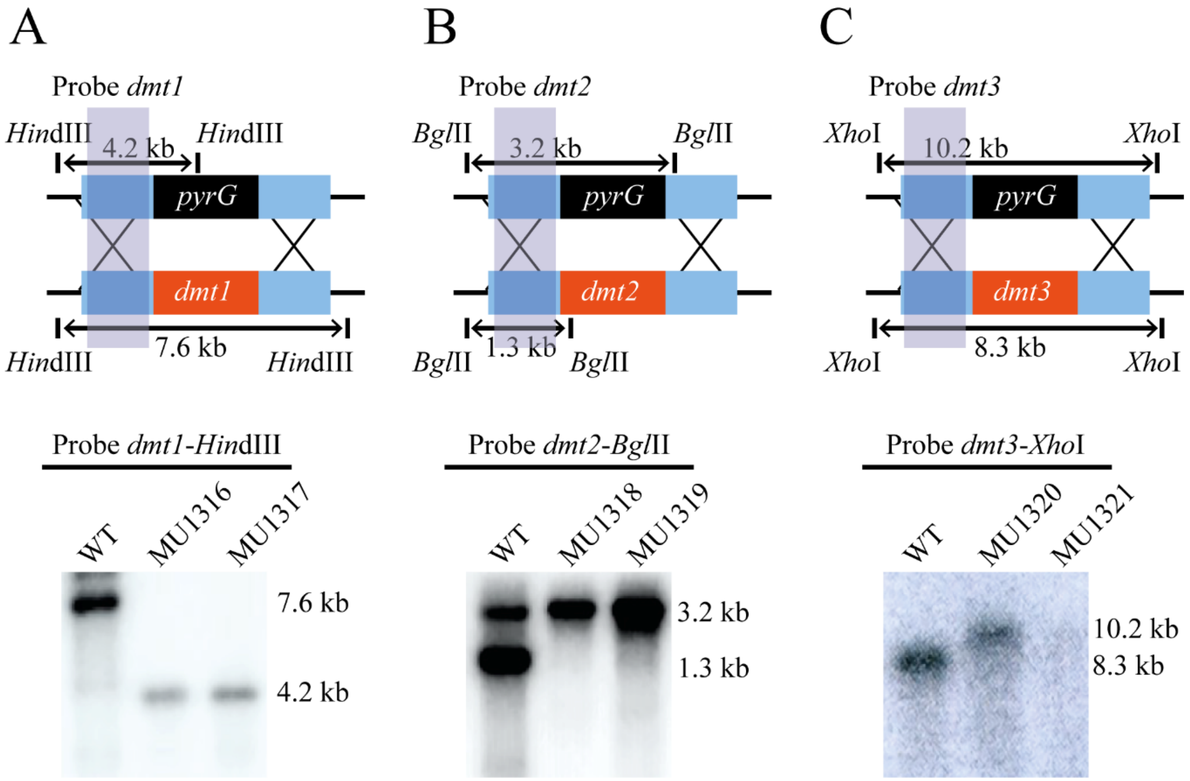

Figure S1. Gene replacement by homologous recombination of the *dmt* genes in *M. lusitanicus*; A) *dmt1*, B) *dmt2*, C) *dmt3*. The recombinant fragments for deletion of each *dmt* gene include the *pyr*G selective marker flanked by 5′and 3′ends of the corresponding gene. The constructs designed were used to replace wild-type locus for each gene by homologous recombination resulting in the mutant locus shown in the upper part of schemes A, B, and C. The cutting sites of restriction enzymes used in Southern-blot hybridization and the sizes of restriction fragment that hybridized with the specific probes are indicated in diagrams A, B, and C. Gel images below each diagram show the results of the hybridization of the genomic DNA from wild type (WT) and mutant strains digested with corresponding restriction enzymes and hybridized with the specific probes for each locus.
