# Supplementary material for: DNA Methylation on N6-Adenine Regulates the Hyphal Development during Dimorphism in the Early-Diverging Fungus Mucor lusitanicus
Source: J Fungi (Basel). 2021 Sep 8;7(9):738. doi: 10.3390/jof7090738 (PMC8470550; doi:10.3390/jof7090738)
Supplement: Supplementary file 1 [file jof-07-00738-s001.zip › jof-1330263-supplementary/jof-1330263-revised/Supplementary Information_Osorio-Concepcion et al_Figure S2.pdf]

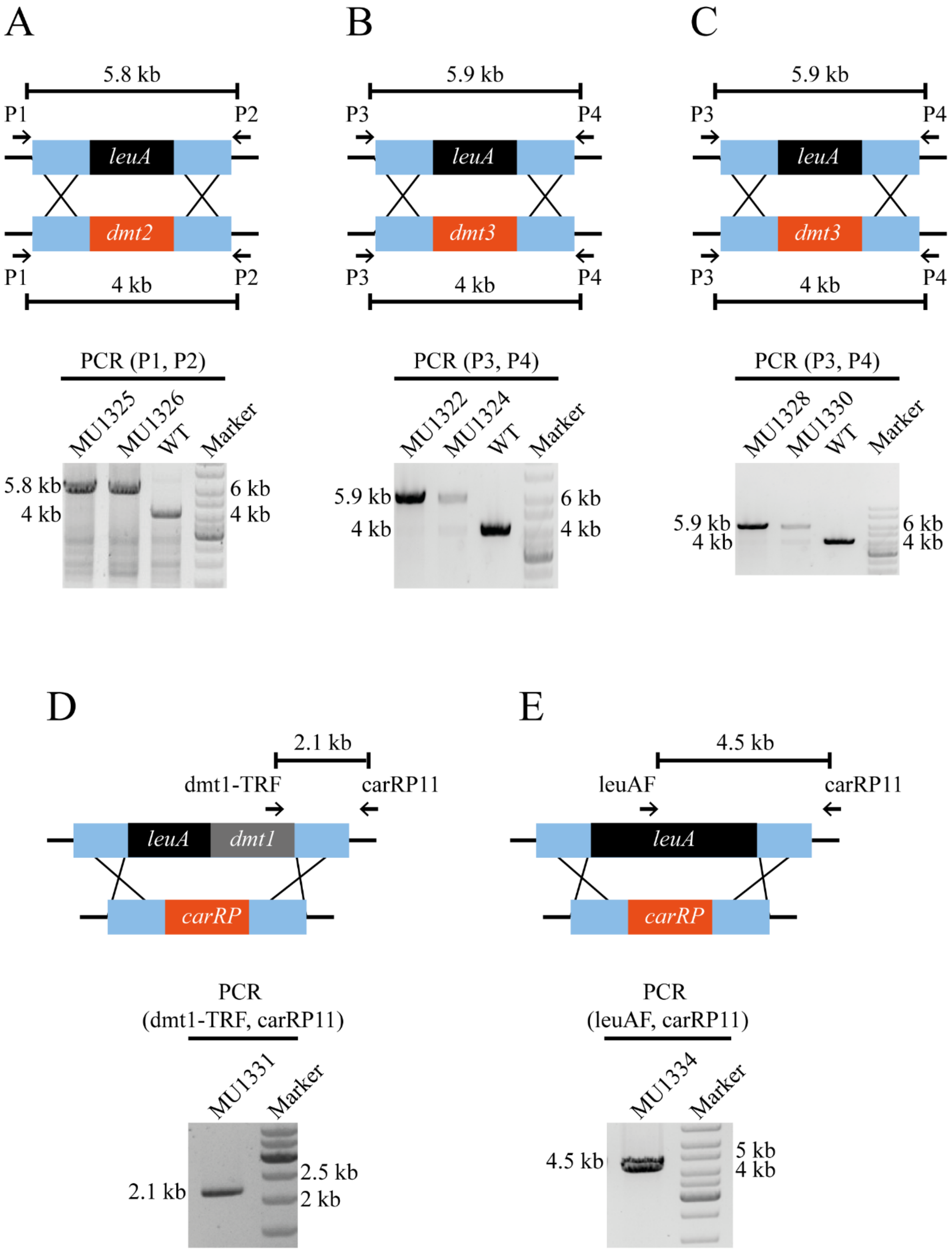

Figure S2. Generation of *dmt* double mutants and complementation of strain MU1317 (*dmt1*Δ) with *dmt1* wild-type allele. A, B, and C) The illustrations represent the mutant locus resulting (upper part) from the double homologous recombination between the wild-type locus and the disruption fragments of *dmt2* and *dmt3* genes. Each fragment consisted of a *leu*A selection marker surrounded by 5′and 3′ends of respective genes and was

used for genetic transformation of single mutants MU1317 (*dmt1*Δ, *leu*A^{-}) (A and B) and MU1319 (*dmt2*Δ, *leu*A^{-}) (C). Gel images below each scheme show the PCR results using genomic DNA from the indicated strains and depicted primers that hybridized upstream and downstream of the integration site. The mobility of the marker fragments were slightly higher than expected. D and E) Schemes of the integration of *dmt1* wild-type allele in the mutant MU1317 (*dmt1*Δ). The deletion fragments containing the *leuA* cassette and *dmt1* gene (D) or only the *leuA* selection marker (E) were integrated into the *carRP* locus by double homologous recombination. The complementation with or without *dmt1* wild-type allele was validated by PCR using primer binding sequences inside and outside the deletion cassette. The binding sites of oligonucleotides for each locus and the size PCR products are shown in schemes (A, B, C, D, and E). The size of wild-type and mutant alleles are indicated below in the diagrams (A, B, C, D, and E).
