# Supplementary material for: DNA Methylation on N6-Adenine Regulates the Hyphal Development during Dimorphism in the Early-Diverging Fungus Mucor lusitanicus
Source: J Fungi (Basel). 2021 Sep 8;7(9):738. doi: 10.3390/jof7090738 (PMC8470550; doi:10.3390/jof7090738)
Supplement: Supplementary file 1 [file jof-07-00738-s001.zip › jof-1330263-supplementary/jof-1330263-revised/Supplementary Information_Osorio-Concepcion et al_Figure S3.pdf]

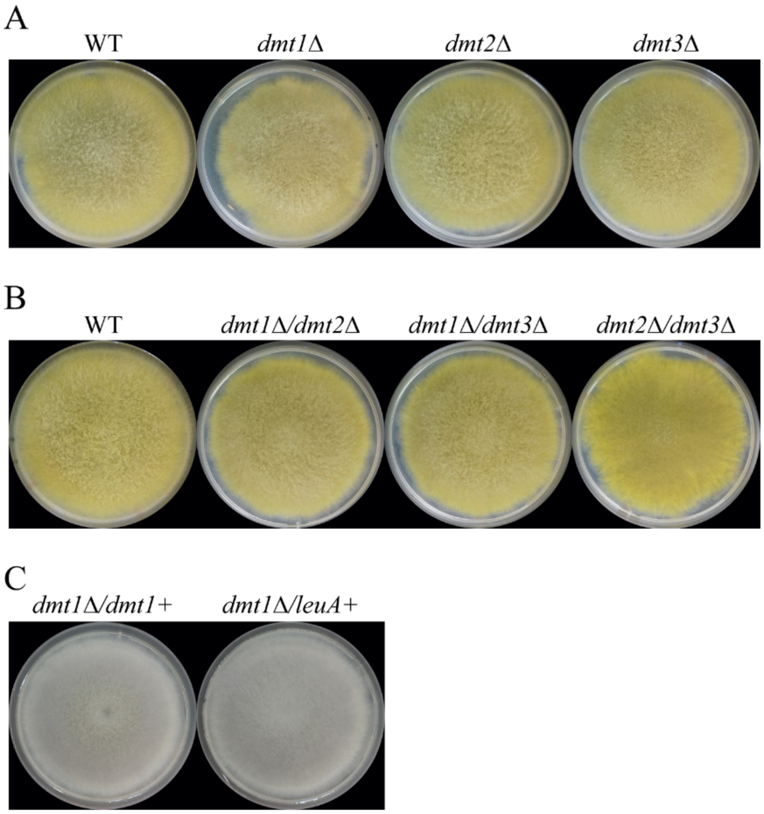

Figure S3. Phenotypic analysis of *dmt* mutants and complemented strains. Small pieces of mycelium from the wild-type strains (WT) MU636 (A) and CBS277.49 (B), single deletion mutants (A), double deletion mutants (B), strains complemented with *dmt1* gene (MU1331) or containing only the *leu*A marker (MU1334) were inoculated at the center of the plate containing solid YPG medium, pH 4.5, and grown for 96 h at 26°C. Strains complemented in C were albino due to the deletion of the *carRP* locus after homologous recombination with the complementation cassettes
