# Supplementary material for: DNA Methylation on N6-Adenine Regulates the Hyphal Development during Dimorphism in the Early-Diverging Fungus Mucor lusitanicus
Source: J Fungi (Basel). 2021 Sep 8;7(9):738. doi: 10.3390/jof7090738 (PMC8470550; doi:10.3390/jof7090738)
Supplement: Supplementary file 1 [file jof-07-00738-s001.zip › jof-1330263-supplementary/jof-1330263-revised/Supplementary Information_Osorio-Concepcion et al_Figure S4_Revised.pdf]

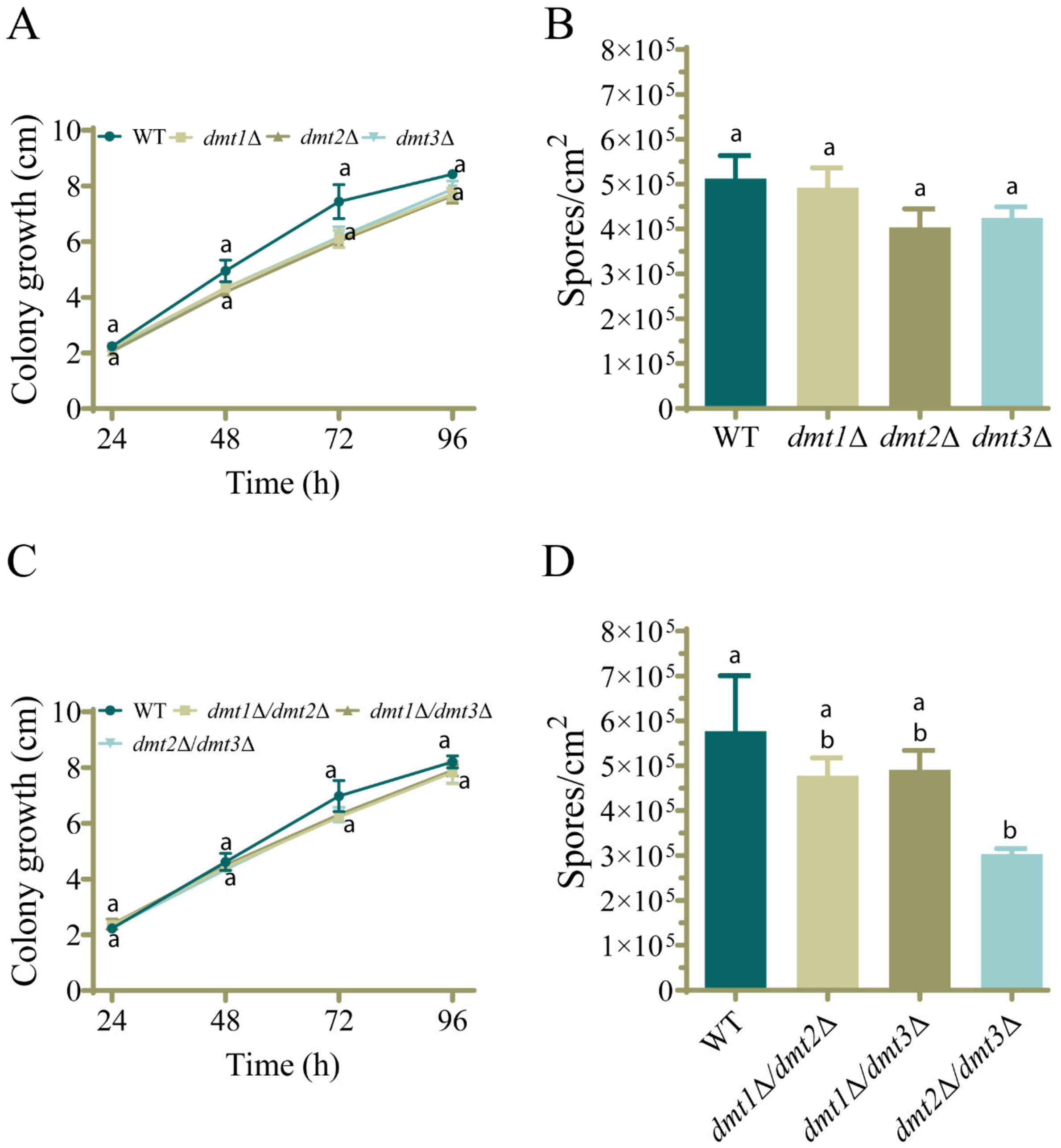

Figure S4. Single and double deletion mutants in the *dmt* genes are not affected in the growth and sporulation. A and C) A drop containing 500 spores of the wild-type (WT), *dmt1Δ* (MU1317), *dmt2Δ* (MU1318), *dmt3Δ* (MU1320), *dmt1Δ/dmt2Δ* (MU1326), *dmt1Δ/dmt3Δ* (MU1326), and *dmt2Δ/dmt3Δ* (MU1330) strains were inoculated on the centre of YPG medium plates and the diameter was measured every 24 h . B and D) Spore production of each strain cultured on solid YPG medium, pH 4.5, was determined by counting the spore number harvested 96 h of growth in 12 h light/dark cycles at 26°C. The graphs display means ± SD. Three independent experiments were conducted for each strain. The wild-type strains are MU636 (A and B) and CBS277.49 (C and D). The statical analysis was performed using ANOVA ($P < 0.001$, Tukey test) and the identical letters mean not significant differences,
