# Supplementary material for: DNA Methylation on N6-Adenine Regulates the Hyphal Development during Dimorphism in the Early-Diverging Fungus Mucor lusitanicus
Source: J Fungi (Basel). 2021 Sep 8;7(9):738. doi: 10.3390/jof7090738 (PMC8470550; doi:10.3390/jof7090738)
Supplement: Supplementary file 1 [file jof-07-00738-s001.zip › jof-1330263-supplementary/jof-1330263-revised/Supplementary Information_Osorio-Concepcion et al_Figure S5.pdf]

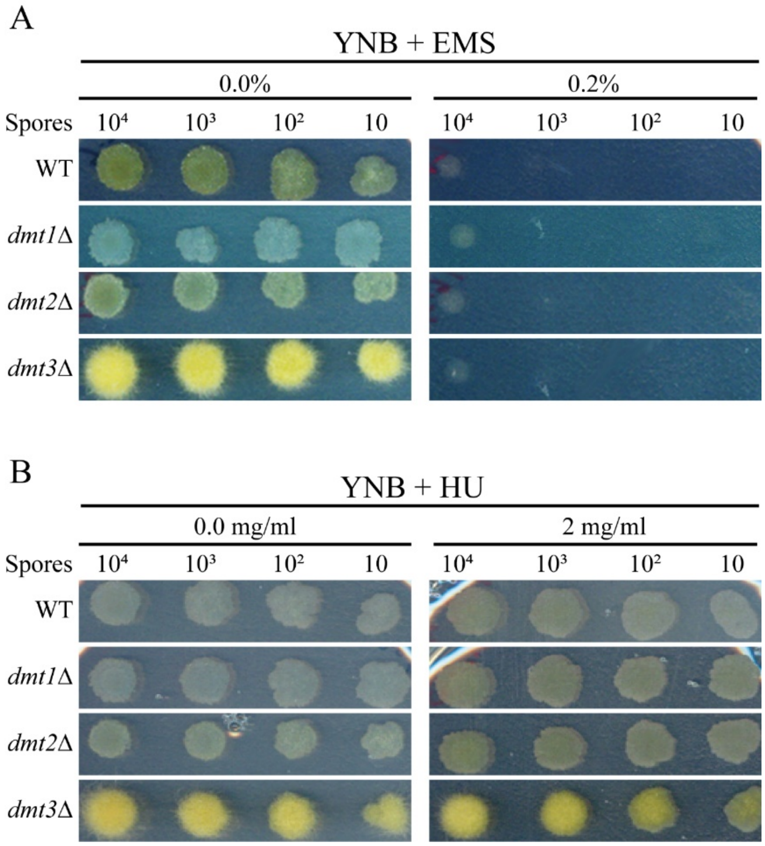

Figure S5. Phenotypic analysis of single deletion mutants in the *dmt1*, *dmt2*, and *dmt3* genes exposed to alkylating agents and compounds inhibiting DNA synthesis. The indicated amount of fresh spores of wild-type strain MU636 (WT) and *dmt* mutants were spotted on YNB agar plates, pH 3, amended with 0.2% EMS (A) or 2 mg/ml HU (B), and grown for 48 h at 26°C. Three independent experiments wer°e conducted for each strain.
