# Supplementary material for: DNA Methylation on N6-Adenine Regulates the Hyphal Development during Dimorphism in the Early-Diverging Fungus Mucor lusitanicus
Source: J Fungi (Basel). 2021 Sep 8;7(9):738. doi: 10.3390/jof7090738 (PMC8470550; doi:10.3390/jof7090738)
Supplement: Supplementary file 1 [file jof-07-00738-s001.zip › jof-1330263-supplementary/jof-1330263-revised/Supplementary Information_Osorio-Concepcion et al_Figure S6.pdf]

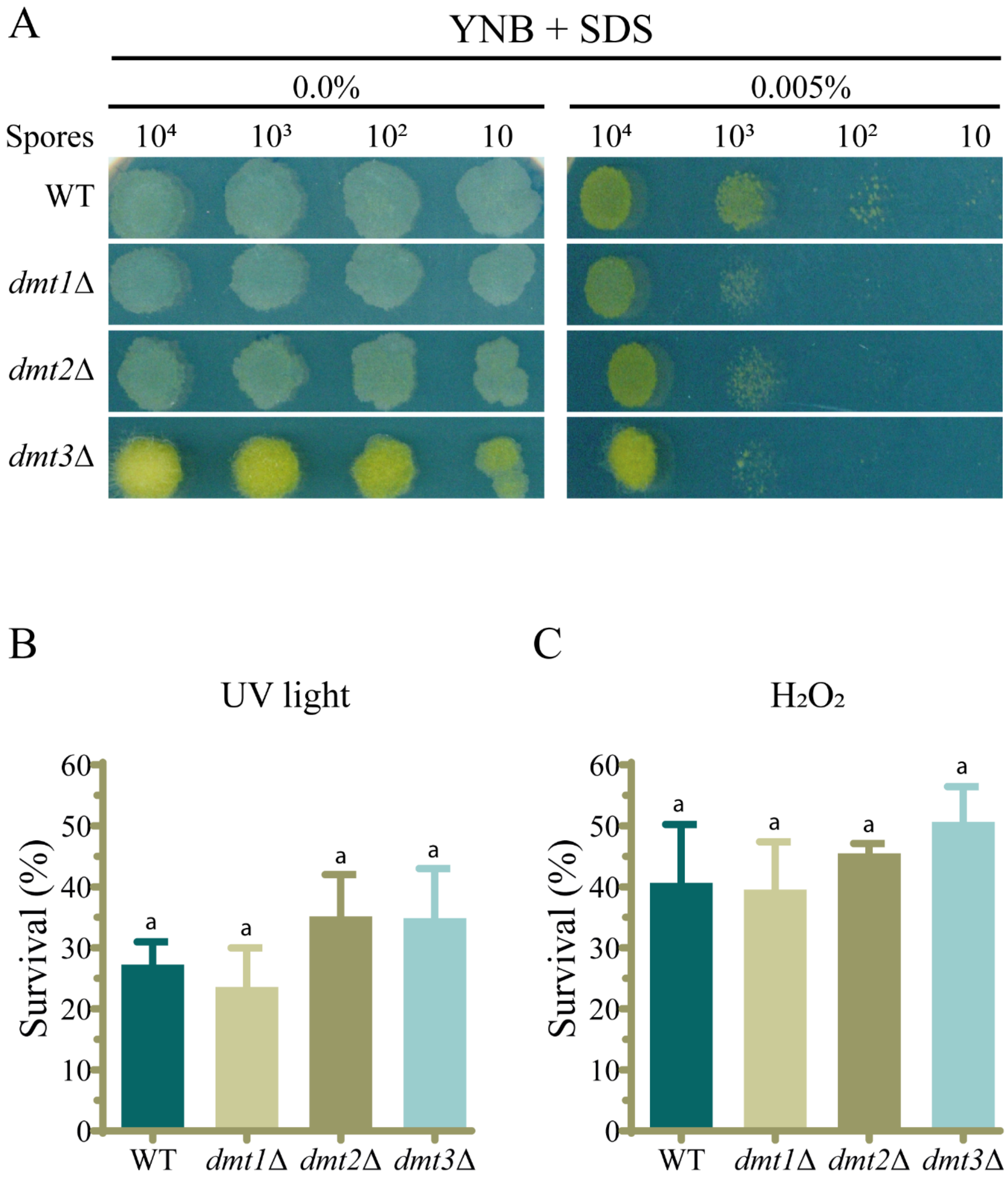

Figure S6. Survival analysis of single deletion mutants in the *dmt1*, *dmt2*, and *dmt3* genes exposed to stress generating compounds and DNA damaging agents. A) Different amounts of fresh spores *dmt* knockout mutants and the wild type strain MU636 (WT) were spotted on YNB agar plates, pH 3, amended with 0.005% SDS, and grown for 48 h at 26°C. B y C) For UV-associated assays and Hydrogen peroxide (H_2O_2), 200 spores were seeded on YNB plates, pH 3, supplemented with exposed to 10 mJ/cm^2 UV, or 5 mM H_2O_2, respectively. All the cultures were incubated for 48 h at 26°C, and the ability to develop colonies and the survival percentage were determined. The graphs display means ± SD. Three independent experiments were conducted. The statical analysis was performed using ANOVA ($P < 0.001$, Tukey test) and the identical letters mean not significant differences.
