# Supplementary material for: DNA Methylation on N6-Adenine Regulates the Hyphal Development during Dimorphism in the Early-Diverging Fungus Mucor lusitanicus
Source: J Fungi (Basel). 2021 Sep 8;7(9):738. doi: 10.3390/jof7090738 (PMC8470550; doi:10.3390/jof7090738)
Supplement: Supplementary file 1 [file jof-07-00738-s001.zip › jof-1330263-supplementary/jof-1330263-revised/Supplementary Information_Osorio-Concepcion et al_Table S1.pdf]

Supplementary Table S1. Mutants generated in this study

Name	Genotype
MU1316	*dmt1Δ::pyrG*$^{+}$, *leuA*$^{-}$
MU1317	*dmt1Δ::pyrG*$^{+}$, *leuA*$^{-}$
MU1318	*dmt2Δ::pyrG*$^{+}$, *leuA*$^{-}$
MU1319	*dm2Δ::pyrG*$^{+}$, *leuA*$^{-}$
MU1320	*dmt3Δ::pyrG*$^{+}$, *leuA*$^{-}$
MU1321	*dmt3Δ::pyrG*$^{+}$, *leuA*$^{-}$
MU1322	*dmt1Δ::pyrG*$^{+}$, *dmt3Δ::leuA*$^{+}$
MU1324	*dmt1Δ::pyrG*$^{+}$, *dmt3Δ:: leuA*$^{+}$
MU1325	*dmt1Δ::pyrG*$^{+}$, *dmt2Δ::leuA*$^{+}$
MU1326	*dmt1Δ::pyrG*$^{+}$, *dmt2Δ::leuA*$^{+}$
MU1328	*dmt2Δ::pyrG*$^{+}$, *dmt3Δ:: leuA*$^{+}$
MU1330	*dmt2Δ::pyrG*$^{+}$, *dmt3Δ::leuA*$^{+}$
MU1331	*dmt1Δ::pyrG*$^{+}$, *carRPΔ::leuA*$^{+}$, *dmt1*$^{+}$
MU1334	*dmt1Δ::pyrG*$^{+}$, *carRPΔ::leuA*$^{+}$
