# Supplementary material for: DNA Methylation on N6-Adenine Regulates the Hyphal Development during Dimorphism in the Early-Diverging Fungus Mucor lusitanicus
Source: J Fungi (Basel). 2021 Sep 8;7(9):738. doi: 10.3390/jof7090738 (PMC8470550; doi:10.3390/jof7090738)
Supplement: Supplementary file 1 [file jof-07-00738-s001.zip › jof-1330263-supplementary/jof-1330263-revised/Supplementary Information_Osorio-Concepcion et al_Table S2.pdf]

Supplementary Table S2. Primers used in this work.

Primer name	Sequence (5’-3’) [a. b]	Experiment
leuAF	TGCTCCCTACCAACTCTATTC	Selective marker amplification, PCR validation
leuAR	GTCGAGTTGACCAGAATGTAC	Selective marker amplification
dmt2-F5’	GCCTGCACGGTATACTGACG	*dmt2* deletion construct
dmt2-R5’+ leuA	GAATAGAGTTGGTAGGGAGCATCAAGAAGGTGGAGGAGAAACA	*dmt2* deletion construct
dmt2-F3’+ leuA	GTACATTCTGGTCAACTCGACAGCATAGTAAAAGATAGATGGAGCGT	*dmt2* deletion construct
dmt2-R3’	CACGCGGAATCTGAATGACG	*dmt2* deletion construct
dmt3-F5	CGGTTGCCCTTTTCTTTCTCG	*dmt3* deletion construct
dmt3-R5+leuA	GAATAGAGTTGGTAGGGAGCATCTCTTTGTGATGAAGGTTGGA	*dmt3* deletion construct
dmt3-F3+leuA	GTACATTCTGGTCAACTCGACAGCCCATTTTGTGATTTGAGGT	*dmt3* deletion construct
dmt3-R3	GTCCCGGTAAGTACAACTGCA	*dmt3* deletion construct
pkaR4-TR-F	AGTCTGGCCTTGCCGTGTTT	RT-qPCR
pkaR4-TR-R	GCAACGACCGTAGCTGCTCT	RT-qPCR
dmt1-F+ XhoI	TCCCTCGAGAGGTCCCCCTTGACACGTGGTTC	*dmt1* ORF
dmt1-R+SacII	TCACCGCGGAGTGCACCATTGGATTCCTTGTCC	*dmt1* ORF
P1	GAAAGCCTGCATTGGATTGGC	*dmt2* replacement confirmation
P2	CGAAAAGTGGCCCCTATCGG	*dmt2* replacement confirmation
P3	GTACACTGTTGCTCTCCATGTG	*dmt3* replacement confirmation
P4	AGCAAATCGTCGTGGATGGT	*dmt3* replacement confirmation
pyrGF	TGCCTCAGCATTGGTACTTG	Selective marker amplification
pyrGR	GTACACTGGCCATGCTATCG	Selective marker amplification
85076 F	ACAATCTCGGCCTCGGAACAAGTGC	*dmt1* deletion construct
85076 RpyrG	CAAGTACCAATGCTGAGGCATGCACCTTGCTGTCATGGCAACAAGC	*dmt1* deletion construct
85076 FpyrG	CGATAGCATGGCCAGTGTACGCTTGGACCAATGGATACCTACTGAC	*dmt1* deletion construct
85076 R	ATCCCCCTTGACACGTGGTTCGTAGC	*dmt1* deletion construct
115786 F	CTCCATGCTGATATCTGTACGCTGT	*dmt3* deletion construct
115786 RpyrG	CAAGTACCAATGCTGAGGCATGAGGTTCAGCTGTGATTTGGCTCCA	*dmt3* deletion construct
115786 FpyrG	CGATAGCATGGCCAGTGTACTGCTGCTGGTGATTGACAGTTGCTG	*dmt3* deletion construct
115786 R	GATGGCTACTGACAAGATCGTTGCTG	*dmt3* deletion construct
106998 F	AGAGCACGCGGAATCTGAATGACG	*dmt2* deletion construct
106998 RpyrG	CAAGTACCAATGCTGAGGCAGTCTATCCACGACATCAGAGCGAGGA	*dmt2* deletion construct
106998 FpyrG	CGATAGCATGGCCAGTGTACCGAAAATAGTGTGCGCCAGCAGGATG	*dmt2* deletion construct

106998 R	CTTGTGGACATGGAAGATGCTGCT	*dmt2* deletion construct
CarRP11	CATACAAAGCACGAGTTTCC	PCR validation
dmt1-TR-F	TGCCTCGTAAACCCAACCACT	PCR validation
qPCR-EF1-F	GTCCGTGATATGCGTCAAACC	RT-qPCR
qPCR-EF1-R	AGCGGCCTTGGTGACCTTAC	RT-qPCR
Udmt1F	ACAATCTCGGCCTCGGAACAAGTGC	Southern blot validadtion
Ddmt1-pyrGR	CAAGTACCAATGCTGAGGCATGCACCTTGCTGTCATGGCAACAAGC	Southern blot validadtion
Udmt2F	AGAGCACGCGGAATCTGAATGACG	Southern blot validadtion
Udmt2-pyrGR	CAAGTACCAATGCTGAGGCAGTCTATCCACGACATCAGAGCGAGGA	Southern blot validadtion
Udmt3F	CTCCATGCTGATATCTGTACGCTGT	Southern blot validadtion
Ddmt3-pyrGR	CAAGTACCAATGCTGAGGCATGAGGTTCAGCTGTGATTTGGCTCCA	Southern blot validadtion

[a] Underlined sequences are complementary to selection marker *pyrG* or *leuA*

[b] Bold bases corresponding to the cutting sites to restriction enzymes
